# Breastfeeding Protects from Overjet in Adolescence by Reducing Pacifier Use: A Birth Cohort Study

**DOI:** 10.3390/nu15153403

**Published:** 2023-07-31

**Authors:** Carlos Alberto Feldens, Laura Boianovsky Petracco, Gustavo G. Nascimento, Huihua Li, Márcia Regina Vítolo, Karen Glazer Peres

**Affiliations:** 1 Graduate Program in Dentistry, Univesidade Luterana do Brasil, Canoas 92425-020, Brazil; cafeldens@gmail.com (C.A.F.); laura.petracco@hotmail.com (L.B.P.); 2 National Dental Research Institute Singapore, National Dental Centre Singapore, Singapore 168938, Singapore; ggn@duke-nus.edu.sg (G.G.N.); li.huihua@duke-nus.edu.sg (H.L.); 3 Oral Health Academic Programme, Duke-NUS Medical School, Singapore 169857, Singapore; 4 Graduate Program in Pediatrics—Child and Adolescent Health Care, Universidade Federal de Ciências da Saúde de Porto Alegre, Porto Alegre 90050-170, Brazil; marciavitolo@hotmail.com

**Keywords:** breastfeeding, pacifier, cohort studies, causal inference, malocclusion

## Abstract

Increased dental overjet in adolescence is a clinically relevant outcome associated with the complexity and high cost of treatment, indicating the need for prevention strategies. We investigated the long-term impact of breastfeeding and pacifier use on increased overjet (IOVJ) in permanent dentition. A prospective cohort nested in a randomized controlled trial was conducted from birth to 12 years of age (*n* = 214). Breastfeeding and pacifier use were recorded monthly until 12 months. Overjet was assessed at age 12 years. We employed a causal mediation analysis using parametric regression models assuming no interaction between breastfeeding and pacifier usage. We found a total protective effect of breastfeeding on IOVJ (OR 0.49; 95% CI 0.28–0.96), where 63.1% were mediated by pacifier use (OR 0.61; 95% CI 0.44–0.87). Breastfeeding directly decreased the odds of IOVJ by 20%; however, the confidence interval included the null estimate (OR 0.81; 95% CI 0.41–1.60). In conclusion, breastfeeding protects by half of the IOVJ in adolescence through reducing pacifier use. Oral and general health professionals should collaborate to support WHO breastfeeding guidelines during individual patient counseling. Guidelines for practice, policy or public information require messages that include a common risk approach to oral and general health.

## 1. Introduction

Increased overjet, a developmental disorder in the craniofacial structures characterized by an exaggerated overlap of the upper incisors [1,2], negatively impacts children, adolescents, and adults’ oral health-related quality of life [3,4,5]. Physical disability, functional limitations, and social well-being represent the quality-of-life domains significantly affected by increased overjet [4,5,6]. Among different types of malocclusions, increased overjet stands out as the most associated adolescent bullying [7]. This form of aggression and violence is related to the visible aesthetic impact caused by increased overjet [8] occurring mainly in the school context, making it difficult for adolescents to integrate into the social environment [9]. Furthermore, a systematic review including more than 50 worldwide primary studies shows that large overjet is the only consistently reported risk factor for traumatic dental injuries, accounting for over two hundred million injuries to anterior teeth [10].

The significant impact of increased overjet on clinically relevant outcomes associated with the complexity and high cost of treatment [11,12] indicates the need for prevention strategies focused on its causal chain. Although genetically influenced, increased overjet, compared to other malocclusions, may respond more to environmental factors, such as the lack of breastfeeding, pacifier use, mouth breathing, and allergic rhinitis [13]. Mainly, breastfeeding may play an essential role in preventing increased overjet due to its ability to promote adequate growth and development of the muscles and bones of the dental arches [14]. Previously, breastfeeding duration over six months was identified as a protective factor for primary-dentition increased overjet in preschool children [14,15]. Pacifier use, on the opposite, can impair the adequate growth of oral structures in the first years of life, representing a risk factor for increased overjet in a similar age group [16]. To date, we have not identified studies investigating the simultaneous and long-term impact of breastfeeding and pacifier use on the occurrence of increased overjet affecting permanent dentition. Moreover, breastfeeding and pacifier use are strongly associated [17,18], thus making their relationship with increased overjet even more complex.

From an analytical perspective, conventional regression analysis tends to fail to account for such a complex scenario in which breastfeeding protects against pacifier use, and its interruption may lead to pacifier initiation [17,19]. To avoid a potential misleading association induced by collider bias, a source of bias often introduced by controlling for a variable in the causal path between the exposure and the outcome, i.e., mediator, researchers may face challenges in operationalizing this intricate relationship [20]. Taking into consideration the mediating role of pacifier use in the path between breastfeeding and increased overjet, mediation analysis using the counterfactual approaches of direct, i.e., effect of breastfeeding only, and indirect, i.e., effect of breastfeeding via pacifier use, effects can be instrumental to further clarifying this relationship and exploring other potential benefits of breastfeeding in addition to preventive strategies to increased overjet. However, thus far, there is a lack of studies using such an approach, leaving a gap to be filled in by this study.

Accordingly, the present birth cohort study aimed to investigate the long-term impact of breastfeeding and pacifier use during early childhood on increased overjet in adolescence. We hypothesized that breastfeeding protects from the risk of increased overjet in permanent dentition.

## 2. Materials and Methods

### 2.1. Setting, Study Design, and Participants

The present study is a prospective cohort nested in a randomized clinical trial (registered with ClinicalTrials.gov; number NCT00629629) in which 500 children were recruited at birth from the maternity of the only public hospital in the city of São Leopoldo (Southern Brazil) from October 2001 to June 2002 [21,22]. São Leopoldo has a population of approximately 213,000 inhabitants, and all homes have access to the fluoridated public water supply from the time the children were born (average 0.7 ppm F). The intervention study aimed to investigate the effectiveness of nutritional counseling during the first year of life on different health outcomes.

After childbirth, mothers who met the inclusion criteria were invited to participate in the study: full-term pregnancy (≥37 weeks) who had children with a normal birth weight (≥2500 g), and no impediment to breastfeeding (i.e., HIV/AIDS). Exclusion criteria were congenital malformations and twin pregnancies.

**Figure 1:**
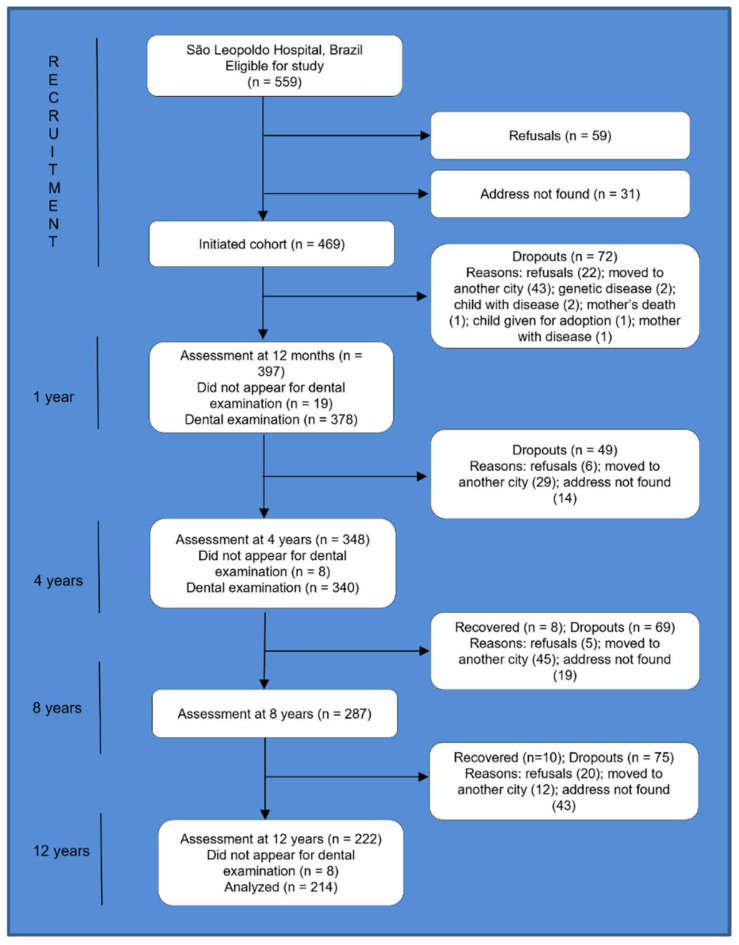
Flow chart of the birth cohort study.

After being informed of the study procedures, 90% of the mothers agreed to participate. All children were assessed at 6 months and 12 months, with further follow-up at 4, 8, and 12 years of age. The present study used data collected at birth and at 1 and 12 years of age. Three hundred ninety-seven children were assessed at 1 year of age. The number of participants recruited and retained at 12 years of age (*n* = 214) determined the sample size for the present cohort study. Finding an outcome proportion (increased overjet) equal to 28.5% among those exposed (breastfeeding up to 12 months) and 47% among those not exposed would be necessary to detect a significant difference (α = 5% and β = 80%) between groups in this study (*n* = 214). Figure 1 displays the cohort flow diagram.

### 2.2. Data Collection

The following data were collected soon after childbirth: child’s sex and anthropometric data (birth weight in grams and length and head circumference in centimeters), family income was collected in Brazilian currency, divided by the Brazilian monthly minimum wages (1BMMW= USD 80.00) and subsequently categorized in <2, 2–3, and >3, mother’s age (years), and mother’s educational attainment in numbers of years of school completed and dichotomized in ≤8 and >8 years.

Nutrition undergraduate students not involved in the intervention program conducted face-to-face structured interviews with the mothers in their homes when their children were 6 and 12 months old. Dietary behavior variables were assessed at 6 and 12 months, using face-to-face structured interviews of the feeding practices’ beginning, duration, and frequency of during the previous six months. These interviews investigated, separately for each month, breast- and bottle-feeding during the day and night (yes/no). The 6- and 12-month interviews also explored, separately for each month, the use of a pacifier (yes/no). For this purpose, parents were asked whether the child had used a pacifier and, in case of a positive answer, the age of starting and stopping its use.

The questionnaires were tested in a pilot study of 16 mothers of children aged 6 and 12 months attending primary care services and modified accordingly. Fieldworkers were submitted to a training program (12 h), which included simulated questions and answers using a standardized questionnaire. Moreover, supervision during fieldwork and data checking by an experienced nutritionist were performed to ensure the methodological quality of data collection.

At 12 years of age, the participants underwent a clinical examination performed by a single calibrated examiner with the child seated on a standard chair. The teeth were brushed and dried with gauze. Each tooth surface was examined with a mouth mirror and a WHO probe under artificial light. The teeth were examined for dental caries [1] and malocclusion using the Dental Aesthetic Index (DAI) [1]. Maxillary overjet was obtained from the DAI index in which it is measured as the distance in mm between the palatal surface of the most protruded maxillary incisor and the labial surface of the corresponding mandibular incisor, the value closest to an integer being recorded. Maxillary overjet was later categorized into normal (≤3.0 mm) or increased (>3.0 mm) [10]. Intra-examiner reproducibility of maxillary overjet measurement was previously tested with two examinations of 20 adolescents and a seven-day interval between sessions (Intraclass Correlation Coefficient = 0.93; 95% CI 0.83–0.97).

### 2.3. Data Analysis

Means with their respective standard deviations were reported for continuous variables, while frequencies followed by their proportion and 95% confidence intervals were reported for categorical data.

**Figure 2:**
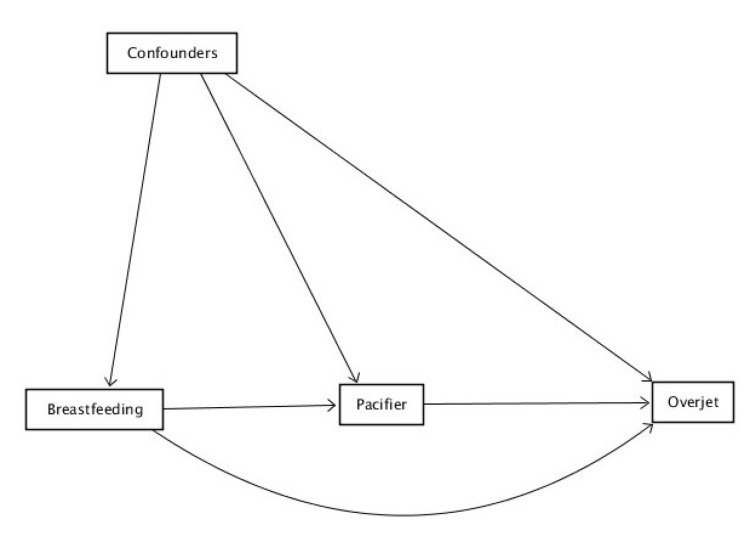
Directed acyclic graph to depict the relationship between breastfeeding (≥12 months) and overjet.

To statistically quantify the extent to which the effect of breastfeeding (exposure) on overjet (outcome) was mediated by the use of a pacifier after accounting for measured confounders (Figure 2), we employed a causal mediation analysis based on the counterfactual outcome framework using parametric regression models [23].

Firstly, we used logistic regression modeling to regress the presence of increased overjet at the age of 12 years on breastfeeding over 12 months, use of a pacifier at 12 months, and the covariates (continuous covariates, including birth weight, length at birth, head circumference at birth, and maternal age at childbirth; categorical covariates, including maternal schooling and income). Secondly, we fitted a logistic regression model to regress pacifier use on breastfeeding at 12 months and the covariates. These procedures were conducted using the CMAverse macro [23]. Briefly, this approach enables the decomposition of the total causal effect of an exposure on an outcome as follows: (i) The natural direct effect (NDE) is due to neither mediation nor interaction, i.e., the effect of breastfeeding on increased overjet, which does not go through pacifier use. To perform the NDE, the exposure is modeled to its counterfactual value while keeping the mediator fixed at whatever it would be for that individual in the absence of exposure (ii) The natural indirect effect (NIE) is due to mediation alone, i.e., the effect of breastfeeding on tooth loss which purely goes through pacifier use. The NIE is calculated by modeling the mediator to its counterfactual value while keeping all individuals under exposure value while keeping the exposure fixed as its baseline value. (iii) The total effect (TE) comprises the sum of previously described direct and indirect effects. Confidence intervals (95% CI) for the effect estimates were obtained via case resampling bootstrapping with 1000 iterations.

Finally, we performed sensitivity analyses using the E-value estimate to test potential unmeasured confounding (U) [24]. The E-value represents the minimum strength of association on the risk ratio scale that an unmeasured confounder would need to have with both the exposure and outcome to eliminate the observed effect of the exposure on the outcome, conditional on the measured covariates. A large E-value would imply that considerable unmeasured confounding would be needed to eliminate the effect estimate. All analyses were performed using R 4.1.1 (https://cran.r-project.org (accessed on 20 July 2023) and STATA 17 (StataCorp., College Station, TX, USA).

## 3. Results

Of 214 participants who provided dental data at 12 years, 201 had completed data at this age. According to Table 1, nearly 60% of the sample was males, with a similar proportion of children whose mothers were between 20 and 30 years when the child was born. Approximately 55% of the children were using a pacifier at 12 months, and a similar proportion had been breastfed for less than 12 months (Table 1). At 12 years, the prevalence of increased overjet was 40% (95% CI 34–46%), significantly higher in adolescents who were not breastfed at 12 months (46.9% versus 32.0%/not breastfed). In addition, the prevalence of increased overjet was significantly higher among those using a pacifier at 12 months (51.4% versus 27.3%/without a pacifier).

**Figure 3:**
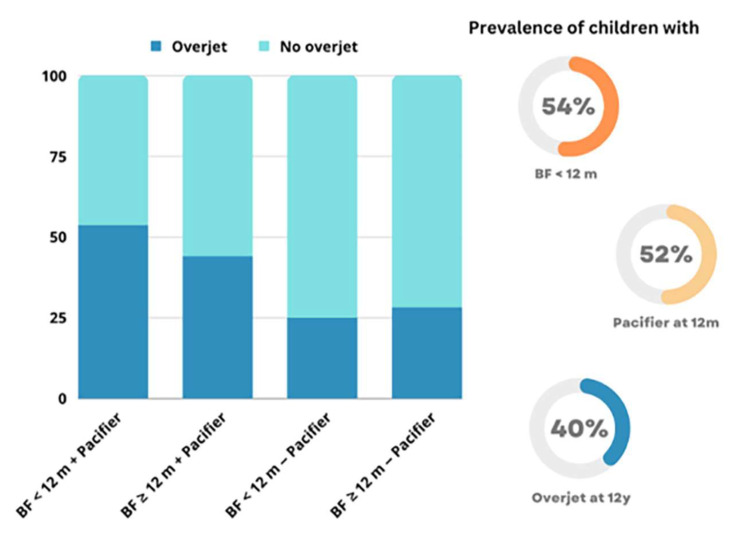
Prevalence of increased overjet at 12 years according to the breastfeeding and pacifier use status at 12 months.

Figure 3 displays the prevalence of increased overjet in permanent dentition according to the breastfeeding and pacifier use status at 12 months. Children who were breastfed for less than 12 months and used a pacifier at 12 months had the highest prevalence of increased overjet. Conversely, children who did not use a pacifier at 12 months had the lowest prevalence of increased overjet, irrespective of their breastfeeding duration.

Table 2 illustrates the results from the effect decomposition analysis. This analysis revealed a total protective effect of breastfeeding on increased overjet (OR 0.49; 95% CI 0.28–0.96; *p*-value 0.039), of which 63.1% were mediated (NIE) by pacifier use (OR 0.61; 95% CI (0.44–0.87; *p*-value 0.005). While breastfeeding directly decreased the odds of increased overjet by 20% (NDE), the confidence interval included the null estimate (OR 0.81; 95% CI 0.41–1.60; *p*-value 0.533).

Sensitivity analysis for unmeasured confounding indicated an E-value of 3.4 (CI 1.2–6.9) for the total effect, suggesting that a three-fold-magnitude association would be required for any unmeasured confounder to eliminate the effect of exposure on the outcome.

## 4. Discussion

Our findings indicated that breastfeeding had an overall protective effect on increased overjet in adolescence, mainly mediated by pacifier use. While breastfeeding did not directly protect against increased overjet, it reduced pacifier use during childhood, lowering the overjet risk in adolescence by half, thus, confirming our hypothesis.

Some specific mechanisms may explain our findings. Sucking requires control of many oral myo-functions, which occur concomitantly with increasing and decreasing pressures of a sucking cycle [25]. In breastfed children, the upward and outward effort of the tongue on the mother’s breasts associated with the coordinated movement of the jaw and facial muscles stimulate the premaxillary region and help the growth and expansion of jaws while flattening the palate [26,27] besides contributing to correctly creating the gateway to the human airways [28,29]. On the other hand, bottle-fed milk requires less tongue pressure and, therefore, less active sucking than breastfeeding. Feeding satiated more quickly through the bottle leads the baby to the continuous need for oral stimulation and comfort provided by using a pacifier [30].

During pacifier use, the posteriorly acting forces of the buccinator muscle act against the forward acting forces of suckling during breastfeeding and may restrain jaw growth [29]. Furthermore, pacifier sucking promotes poor swallow muscle tone interfering with jaw and airway growth which may narrow the roof of the mouth, making it difficult for the child to breathe through the nose. Consequently, an altered breathing pattern through the mouth can be established, resulting in postural, neuromuscular, skeletal, and dental alterations. Essentially, the suction forces of the pacifier during the critical period of postnatal growth can block the potential for genetic growth, in addition to causing changes in the inclination of the upper and lower anterior teeth in a buccal and lingual direction, respectively, establishing an increase in the overjet status.

Pacifier use is also causally associated with weaning. Moreover, it is also a marker of interference with breastfeeding or even if the pacifier is being used as a strategy to start the weaning process [17,18,31,32]. In these cases, the expected protective effect of breastfeeding on the development of the dental arches would be lower than that observed in the absence of this interference.

The benefits of breastfeeding and the negative impact of using a pacifier on different types of malocclusions are well established in primary dentition, with several systematic reviews [14,33,34] supporting such associations in children under six years of age. On the other hand, only a few studies have investigated the relationship between breastfeeding and malocclusion in mixed dentition, a transition phase between the primary and permanent dentitions from 6 to 11 years of age, or in permanent dentition, a period at the beginning of adolescence. Of those, no association was found between breastfeeding and increased overjet in mixed dentition [33,35]. However, the transition phase of mixed dentition is marked by a dynamic of growth and development of the craniofacial complex that may influence the final occlusion pattern. The grey area between primary and permanent dentition concerning the establishment of malocclusions is partially overcome by some studies that show that primary-dentition malocclusion predicts permanent-dentition malocclusion [36,37,38]. However, with the establishment of permanent dentition from the age of 12, the occlusion stabilizes, and self-correction of malocclusions such as increased overjet is no longer expected and may require orthodontic treatment.

As far as the authors’ knowledge, this is the first study to investigate the effect of breastfeeding and the mediation role of a pacifier in permanent dentition. Focusing on specific malocclusion measures plausibly associated with sucking movements may be more revealing in studying breastfeeding and malocclusion [39].

Some methodological aspects of our study merit attention and thus should be properly examined. Firstly, the analytical approach employed in the study allows the estimation of the total, direct, and indirect (via pacifier use) effects of breastfeeding on overjet. Although no statistical significance was found (the confidence interval includes the null value) for the direct effect, the direction of the estimated association suggests a protective effect of breastfeeding on overjet. When analyzing the total effect that comprises direct and indirect effects, there seems to be a direct protective effect of breastfeeding; however, it comprises the null value within 95% confidence interval limits. Therefore, it is not prudent to completely rule out a potentially beneficial direct effect of breastfeeding on overjet. More longitudinal studies with larger samples are needed to examine this matter, although we recognize that longitudinal studies with information on oral health across the life course are rare.

Some potential study limitations merit discussion, such as the high attrition rate over the cohort time. However, the dropout rate is similar to other birth cohort studies [22], and no differences were found between the children of the baseline cohort and those analyzed at 12 years of age [40]. Some degree of outcome measurement bias may also have occurred. However, this possibility is low since overjet size is an objective measurement and easy to obtain. Furthermore, if present, the direction of this bias would be towards the nullity of the association.

A central contribution of our study relates to the observed path linking breastfeeding to increased overjet through pacifier use. To properly interpret our findings, one has to bear in mind the counterfactual approach applied in the study, as well as the concepts of natural direct and indirect effects. The effect decomposition approach currently employed allowed us to identify that the breastfed children at 12 months used pacifiers less than those not breastfed at this age. Consequently, the reduction in pacifier use (influenced by breastfeeding) led to a lower overjet presence at age 12 years. Although the indirect effect accounted for approximately 65% of the total effect, a small, though not significant, direct effect of breastfeeding was noted, as previously discussed. Other methodological aspects of this study, such as its population-based longitudinal design, long follow-up period, and use of clinical data, reinforce the robustness and relevance of our findings. Finally, upon assuming the absence of unmeasured confounding (unlikely to occur as indicated by the E-value estimation) and measurement bias (mitigated by the study design and clinical examinations), our estimates may have a causal interpretation.

From a clinician perspective, we emphasize the importance of oral health professionals, nurses, and paediatricians working together to support WHO breastfeeding guidelines during individual patient counseling. Previously, a randomized controlled trial demonstrated the effectiveness of home visits for advising mothers about breastfeeding and weaning on the reduction of pacifier use in the first year of life [41]. This approach must be accompanied by clarification on the potential risk that using a pacifier causes in the misalignment of the teeth. The reduction in pacifier use can also bring additional benefits to child health, such as improved speech and breathing functions [42]. This information is of direct relevance to promoting activities at the population level. Guidelines for practice, policy or public information require messages that include a common risk approach to oral and general health.

## 5. Conclusions

Breastfeeding reduced the risk of increased overjet in adolescence by half, mainly by decreasing pacifier use in early childhood. The identification of this long-term benefit of breastfeeding in a clinically relevant outcome reinforces the importance of policies to promote breastfeeding and the need for support from health professionals in promoting WHO guidelines during individual patient counselling.

## Figures and Tables

**Table 1:** Total sample distribution and according to the presence of overjet at 12 years.

	Total	Overjet (Yes)	
	Frequency (Proportion)	Frequency (Proportion)	95% CI
Sex			
Male	127 (59.3%)	52 (40.9%)	34.4%, 47.5%
Female	87 (40.7%)	34 (39.1%)	32.5%, 45.6%
Maternal Age			
<20 years old	36 (16.8%)	12 (33.3%)	27.0%, 39.6%
20–30 years old	124 (57.9%)	54 (43.5%)	36.9%, 50.2%
>30 years old	54 (25.2%)	20 (37.0%)	30.6%, 43.5%
Maternal Schooling			
≤8 years	111 (52.1%)	45 (40.5%)	33.9%, 47.1%
>8 years	102 (47.9%)	41 (40.2%)	33.6%, 46.8%
Household monthly income (BMW)			
<2	91 (43.8%)	43 (47.3%)	40.5%, 54.0%
2–3	52 (25.0%)	19 (36.5%)	30.0%, 43.1%
>3	65 (31.2%)	21 (32.3%)	26.0%, 38.7%
Birth Weight (g)			
≤3350	108 (51.2%)	40 (37.0%)	30.5%, 43.6%
>3350	103 (48.8%)	45 (43.7%)	37.0%, 50.4%
Birth Length (cm)			
≤49	133 (63.0%)	57 (42.9%)	36.2%, 49.5%
>49	78 (37.0%)	28 (35.9%)	29.4%, 42.4%
Head Circumference (cm)			
≤35	123 (58.9%)	51 (41.5%)	34.8%, 48.1%
>35	86 (41.1%)	32 (37.2%)	30.7%, 43.8%
Breastfeeding ≥ 12 months			
No	113 (53.8%)	53 (46.9%)	40.2%, 53.7%
Yes	97 (46.2%)	31 (32.0%)	25.7%, 38.3%
Pacifier at age 12 months			
No	99 (47.6%)	27 (27.3%)	18.8%, 37.1%
Yes	109 (52.4%)	56 (51.4%)	41.6%, 61.1%

BMW = Brazilian monthly minimum wage = USD 80.00/month.

**Table 2:** Standardized direct, indirect, and total effects of breastfeeding on increased overjet with pacifier usage at 12 months as the mediator.

	OR (95% CI)	*p*-Value
Natural Direct Effect	0.81 (0.41–1.60)	0.533
Natural Indirect Effect	0.61 (0.44–0.87)	0.005
Total Effect	0.49 (0.28–0.96)	0.039

## Data Availability

The data presented in this study are available on request from the corresponding author. The data are not publicly available due to ethical reasons.
